# Supplementary material for: Efficient single-copy HDR by 5’ modified long dsDNA donors
Source: eLife. 2018 Aug 29;7:e39468. doi: 10.7554/eLife.39468 (PMC6125127; doi:10.7554/eLife.39468)
Supplement: Supplementary file 1. — Embryos injected with unmodified or modified (5’Biotin, Amino-dT, Spacer C3) long dsDNA gfp donor cassettes matching the rx2, actb, dnmt1 or rx1 locus, were scored for GFP expression and survival. Injections without Cas9 mRNA for control. [file elife-39468-supp1.docx]

**Supplementary File 1**

| locus | ***rx2*** | | | | | | ***actb*** | | ***dnmt1*** | | ***rx1*** |
| --- | --- | --- | --- | --- | --- | --- | --- | --- | --- | --- | --- |
| modification | - | - | Biotin | Biotin | A-dT | SpC3 | - | Biotin | - | Biotin | Biotin |
| Cas9 | - | + | - | + | + | + | + | + | + | + | + |
| Total injected | 93 | 640 | 133 | 474 | 379 | 395 | 65 | 105 | 94 | 105 | 103 |
| GFP positive | 0 | 41 | 0 | 69 | 29 | 65 | 17 | 33 | 11 | 9 | 12 |
| dead | 3 | 54 | 1 | 38 | 192 | 87 | 15 | 34 | 41 | 4 | 14 |
| %GFP/surviving | 0.00 | 7.00 | 0.00 | 15.83 | 15.51 | 21.10 | 34.00 | 46.48 | 20.75 | 8.91 | 13.48 |
| % death rate | 3.23 | 8.44 | 0.75 | 8.02 | 50.66 | 22.03 | 23.08 | 32.38 | 43.62 | 3.81 | 13.59 |
