# Supplementary material for: Efficient single-copy HDR by 5’ modified long dsDNA donors
Source: eLife. 2018 Aug 29;7:e39468. doi: 10.7554/eLife.39468 (PMC6125127; doi:10.7554/eLife.39468)
Supplement: Supplementary file 2. — Restriction enzyme sites used for cloning of PCR amplicons are indicated in italics. Substituted nucleotides to facilitate T7 in vitro transcription of the sgRNA oligonucleotides are shown in small letters (Stemmer et al., 2015). Locus primers forward (Lf) and reverse (Lr) of respective gene loci, gfp primers forward (Gf) and reverse (Gr), gfp sequencing primers gfpf and gfpr and primers to amplify the mgfp-flexible linker, as well as the gfp probe for Southern Blot analysis, are given. Asterisks indicate phosphorothioate bonds, ‘5’moiety’ was either 5’Biotin, Amino-dT or Spacer C3 in the pDest f mod, pDest r mod, pCS2 f mod and pCS2 r mod primers. [file elife-39468-supp2.docx]

**Supplementary File 2**

| **primer name** | **primer sequence 5’-3’** |
| --- | --- |
| ***rx2*** | |
| rx2 5’HF f  (BamHI) | GCC*GGATCC*AAGCATGTCAAAACGTAGAAGCG |
| rx2 5’HF r  (KpnI) | GCC*GGTACC*CATTTGGCTGTGGACTTGCC |
| rx2 3’HF f  (BamHI) | GCC*GGATCC*CATTTGTCAATGGACACGCTTGGGATGGTGGACGAT |
| rx2 3’HF r  (KpnI) | GCC*GGTACC*TGGACTGGACTGGAAGTTATTT |
| rx2 sgRNA f | TAgGCATTTGTCAATGGATACCC |
| rx2 sgRNA r | AAACGGGTATCCATTGACAAATG |
| rx2 Lf / 5’UTRf | TGCATGTTCTGGTTGCAACG |
| rx2 Lr | AGGGACCATACCTGACCCTC |
| 3’UTRr | GCGACAGCTATTCCACATAAAAAC |
| ***actb*** | |
| actb 5’HF f  (BamHI) | GG*GGATCC*CAGCAACGACTTCGCACAAA |
| actb 5’HF r  (KpnI) | GG*GGTACC*GGCAATGTCATCATCCATGGC |
| actb 3’HF f  (BamHI) | GG*GGATCC*GACGACGATATAGCTGCACTGGTTGTTGACAACGGATCTG |
| actb 3’HF r  (KpnI) | GG*GGTACC*CAGGGGCAATTCTCAGCTCA |
| actb sgRNA f | TAGGATGATGACATTGCCGCAC |
| actb sgRNA r | AAACGTGCGGCAATGTCATCAT |
| actb Lf | GTCCGAGTTGAGGGTGTCTG |
| actb Lr | CATGTGCTCCACTGTGAGGT |
| ***dnmt1*** | |
| dnmt1 5’HF f  (SalI) | AATTT*GTCGAC*GCTTTGACAGTTAACCTACACG |
| dnmt1 5’HF r  (AgeI) | AATTT*ACCGGT*CGTAACTGCAAACTAAAAAATAAAAC |
| dnmt1 3’HF f  (SpeI) | AATTT*ACTAGT*ATGCCATCCAGAACGTCCTTATCTCTACCAGACGATGTCAGAAAAAGGTAC |
| dnmt1 3’HF r  (NotI) | AATTT*GCGGCCGC*CTACACATATTGTCTGTGATAC |
| mgfpf (*AgeI*) | AATTT*ACCGGT*ACTAGTACCATGAGTAAAGGAGAAGAACTTTTCAC |
| mgfpr (*SpeI*) | AATTT*ACTAGT*CGCGGCTGCACTTCCACCGCCTCCCGATCCGCCACCGCCAGAGCCACCTCCGCCTGAACCGCCTCCACCGCTCAGGCTAGCTTTGTATAGTTCATCCATGCCATG |
| dnmt1 sgRNA f | TAgGACATCGTCTGGCAAAGAC |
| dnmt1 sgRNA r | AAACGTCTTTGCCAGACGATGT |
| dnmt1 Lf | CTCAATGTAAACACTTCGTGTCGCTTC |
| dnmt1 Lr | TTGCATGCATATTCAAAGTTGTCAAAG |
| ***rx1*** | |
| rx1 5’HF f  (BamHI) | GCC*GGATCC*GCATCCGAAAGGTAAGGACTGCAAACC |
| rx1 5’HF r  (KpnI) | GCC*GGTACC*CATGAGAGCGTCTGGGCTCTGACC |
| rx1 3’HF f  (BamHI) | GGC*GGATCC*CATTTATCACTCGATACCATGAGCA |
| rx1 3’HF r  (KpnI) | GGC*GGTACC*TTCCAGTTTAAGAACATCCCCTCT |
| rx1 sgRNA1 f | TAggAAATGCATGAGAGCGTCT |
| rx1 sgRNA1 r | AAACAGACGCTCTCATGCATTT |
| rx1 sgRNA2 f | TAggCTCTCATGCATTTATCAC |
| rx1 sgRNA2 r | AAACGTGATAAATGCATGAGAG |
| rx1 Lf | CTTTGCTGTTTTGAGAATTGCACC |
| rx1 Lr | GAGACCGAACGATGACAATAACAC |
| **Backbone primers for *gfp* cassette amplification** | |
| pDest f (control) | CGAGCGCAGCGAGTCAGTGAG |
| pDest r (control) | CATGTAATACGACTCACTATAG |
| pDest f mod | 5’moiety-C*G*A*G*C*GCAGCGAGTCAGTGAG |
| pDest r mod | 5’moiety-C*A*T*G*T*AATACGACTCACTATAG |
| pCS2 f | CCATTCAGGCTGCGCAACTG |
| pCS2 r | CACACAGGAAACAGCTATGAC |
| pCS2 f mod | 5’moiety-C*C*A*T*T*CAGGCTGCGCAACTG |
| pCS2 r mod | 5’moiety-C*A*C*A*C*AGGAAACAGCTATGAC |
| **diagnostic *gfp* primer** | |
| Gf | ATGGCAAGCTGACCCTGAAGTTCATCTGCACCACCGGCAAGC |
| Gr | CTCAGGTAGTGGTTGTCG |
| gfpf | GCTCGACCAGGATGGGCA |
| gfpr | CTGAGCAAAGACCCCAACGAGAAGCGCGATCACATG |
| gfp probe f | GTGAGCAAGGGCGAGGAGCT |
| gfp probe r | CTTGTACAGCTCGTCCATG |
